# Novel Lactone-Based Insecticides and *Drosophila suzukii* Management: Synthesis, Potential Action Mechanisms and Selectivity for Non-Target Parasitoids

**DOI:** 10.3390/insects14080697

**Published:** 2023-08-09

**Authors:** Javier G. Mantilla Afanador, Sabrina H. C. Araujo, Milena G. Teixeira, Dayane T. Lopes, Cristiane I. Cerceau, Felipe Andreazza, Daiana C. Oliveira, Daniel Bernardi, Wellington S. Moura, Raimundo W. S. Aguiar, Ana C. S. S. Oliveira, Gil R. Santos, Elson S. Alvarenga, Eugenio E. Oliveira

**Affiliations:** 1 Research Institute in Microbiology and Agroindustrial Biotechnology, Universidad Católica de Manizales, Carrera 23, 60–63, Manizales 170002, Colombia; jmantilla@ucm.edu.co; 2 Departamento de Entomologia, Universidade Federal de Viçosa (UFV), Viçosa 36570-900, MG, Brazil; andreazzafelipe@yahoo.com.br; 3 Departamento de Química, Universidade Federal de Viçosa (UFV), Viçosa 36570-900, MG, Brazilelson@ufv.br (E.S.A.); 4 Department of Plant Protection, Federal University of Pelotas, Pelotas, Mailbox 354, Capão-do-Leão 96010-900, RS, Brazildbernardi2004@yahoo.com.br (D.B.); 5 Programa de Pós-Graduação em Biodiversidade e Biotecnologia—Rede Bionorte, Universidade Federal do Tocantins (UFT), Gurupi 77402-970, TO, Brazil; 6 Programa de Pós-Graduação em Biotecnologia, Universidade Federal do Tocantins (UFT), Gurupi 77402-970, TO, Brazil; rwsa@uft.edu.br (R.W.S.A.); gilrsan@uft.edu.br (G.R.S.)

**Keywords:** spotted wing *Drosophila*, *Trichopria anastrephae*, in silico approaches, pesticide mode of action

## Abstract

**Simple Summary:**

*Drosophila suzukii* is an insect of global economic importance, including in the Neotropical region. Due to the difficulty in controlling this insect pest with conventional insecticidal molecules, it is necessary to search for novel alternatives. Here, we present the potential of synthetic lactone-based insecticides to control *D. suzukii*. Additionally, we demonstrate molecular predictions regarding the actions of these molecules on the nervous system of the target pest and on the nervous system of its parasitoid, *Trichopria anastrephae*. By using in silico approaches, we demonstrate that the lactone derivatives (*rac*)-8 and compound **4** predominantly affect the TRP channels of *D. suzukii* (TRPM) and exhibit less stable interactions with the TRP channels expressed in *T. anastrephae*.

**Abstract:**

*Drosophila suzukii*, an invasive insect pest, poses a significant threat to various fruit crops. The use of broad-spectrum insecticides to control this pest can reduce the effectiveness of biological control agents, such as the parasitoid *Trichopria anastrephae*. Here, we evaluated the toxicity of newly synthesized lactone derivatives on *D. suzukii* and their selectivity towards *T. anastrephae*. We used in silico approaches to identify potential targets from the most promising molecules in the *D. suzukii* nervous system and to understand potential differences in susceptibilities between *D. suzukii* and its parasitoid. Of the nine molecules tested, (*rac*)-8 and compound **4** demonstrated efficacy against the fly. Exposure to the estimated LC_90_ of (*rac*)-8 and compound **4** resulted in a mortality rate of less than 20% for *T. anastrephae* without impairing the parasitoid’s functional parasitism. The in silico predictions suggest that (*rac)*-8 and compound **4** target gamma amino butyric acid (GABA) receptors and transient receptor potential (TRP) channels of *D. suzukii*. However, only the reduced interaction with TRP channels in *T. anastrephae* demonstrated a potential reason for the selectivity of these compounds on the parasitoid. Our findings suggest the potential for integrating (*rac*)-8 and compound **4** into *D. suzukii* management practices.

## 1. Introduction

The spotted wing drosophila, *Drosophila suzukii*, is a significant insect species that reduces flesh fruit productivity in the Neotropical region [1,2]. Originally from Asia, *D. suzukii* has now spread worldwide [3], and despite its recent invasion into orchards in the Neotropical region, 28 plant species have been identified as hosts for *D. suzukii* [2]. For instance, *D. suzukii* infestation has led to estimated productivity losses of around 30% for Neotropical strawberry production [4].

The control of *D. suzukii* in the Neotropical region, as already described for Europe and the USA [5,6], is heavily dependent on the use of a few molecules (e.g., organophosphates, pyrethroids and the spinosyns) with very-well-characterized undesired effects on non-target organisms, including those that can provide naturally occurring biological control [5,6,7]. A possible alternative to foliar spraying is the use of toxic baits or low-volume, reduced-risk sprays in conjunction with feeding attractants [8,9]. However, although the use of these devices can substantially reduce the amount of insecticide applied, the efficiency can be strongly influenced by factors such as the high density of insects, unharvested fruits, and other alternative host fruits in the field, in addition to the physiological aspects (e.g., reproductive maturity, age, mating status) of insects [10,11].

In support of sustainable control options and compatible production methods of small fleshy fruits, the use of parasitoids has been widely investigated [12,13]. The pupal idiobiont parasitoid *Trichopria anastrephae* has been proposed as an effective biological control agent for *D. suzukii* [2]. This parasitoid is naturally distributed in Brazilian regions with occurrence on blackberry and strawberry fruits attacked by *D. suzukii* [14,15]. It is able to achieve a parasitism rate of over 90% at some sites in Southern France [16]. Thus, generating alternative pesticides compatible with the conservation of beneficial insects can be a robust factor for the control of *D. suzukii* populations. 

Macrocyclic lactones, such as avermectins and milbemycins, have been widely used as insecticides to control a variety of insect pests with reported low risk to non-target insects [17,18,19,20]. These lactones are derived from naturally occurring compounds produced by soil-dwelling bacteria belonging to the genera Streptomyces (for avermectins) and Streptomyces and Streptomyces avermitilis (for milbemycins) [18]. However, there is a knowledge gap regarding the potential modes of action of synthetic lactone derivatives in target and non-target organisms. For instance, previous investigations have demonstrated the actions of some macrocyclic lactones on ligand-activated receptors (e.g., GABA receptors) and transient potential receptor (TRP) channels expressed in invertebrate nervous systems [21,22,23]. Indeed, considering the fact that the differential actions of novel insecticides on GABA receptors and TRP channels have been demonstrated for insect pests and their natural enemies [24,25,26,27], it would be reasonable to expect that such differential activities might be related to the lactone derivatives.

Here, we synthesized novel lactone derivatives and evaluated the toxicity of lactone derivatives on *D. suzukii* and its parasitoid, *T. anastrephae*. We further conducted in silico approaches to identify potential physiological targets in the *D. suzukii* nervous system for the actions of the most promising lactone derivatives. Such molecular prediction approaches helped to assess the action targets with higher selectivity potential for *T. anastrephae*. 

## 2. Materials and Methods

### 2.1. Chemicals and Synthesis Process

We synthesized nine lactone derivatives. The identification of the compounds, as well as their molecular structures, is described in Table S1. The progress of reactions to obtain all the molecules used in this study was monitored by thin-layer chromatography (TLC) plates, and purification was performed by column chromatography on silica gel 70–230 mesh. When necessary, solvents and reagents were purified according to the literature [19]. Complete and detailed synthesis of the molecules is described in Teixeira et al. [28] and Näsman [20].

### 2.2. Chemical Solutions Preparation

The solid crystals of each molecule were weighed in 25 mL scintillation glass vials at masses that would allow the desired concentration (i.e., 1000 mg L^−1^) to be reached after the addition of the solvents, i.e., dimethyl sulfoxide (DMSO) and sugar water solution at 20% *m*/*v*. To dilute the molecules, first, an amount of DMSO that represented 2% of the final volume of the solution, according to the exact molecular mass present in each sample, was added to the vial. The DMSO + molecule mix was then gently hand-stirred preventing the unnecessary spread of the solids on the vial walls. After allowing these first mixes to rest for at least 5 min, or enough for all the crystals solubilize in the DMSO, the remaining volume needed to reach the final solution volume was completed using a prediluted 20% *m*/*v* sugar water solution. The addition of the sugar water into the DMSO + molecule mix must be performed very gently and slowly by releasing the sugar water at the walls of the vial, preventing turmoil or strong disturbance in the solution, followed by gentle stir using a metal spatula. Failure in this step results in the molecule reprecipitating at the bottom or surface of the solution, preventing even exposure to the chemical later on. The control treatment consisted of a solution of 20% *m*/*v* sugar water containing 2% DMSO.

### 2.3. Toxicities on D. suzukii

The toxicity ratios between the compounds were estimated following the methodology proposed by Andreazza et al. [29]. Briefly, the initial assessment of the toxicities of the lactone derivatives in *D. suzukii* adults was conducted by exposing adult flies to a discriminatory concentration of 3 g L^−1^ for a 24 h period. For those lactone derivatives that killed more than 80% at the initial test, we formed concentration–mortality curves. For both the initial discriminatory test and the subsequent concentration–mortality curve assays, the exposure was completely randomized. Our experimental unit consisted of 25 unsexed 3–4-day-old flies placed into a 250 mL glass vial. To prepare each exposure unit, a dental cotton wick was placed inside a 250 mL glass vial, and 1.8 mL of the testing solution was applied to the cotton wicks. Subsequently, the vial was closed at the top with a foam plug. The fly release occurred by inserting a plastic tube between the plug and the vial’s wall and puffing the flies into the vial. The insects could then feed on the solution ad libitum. At the end of 24 h period, the mortality was checked, and a fly was considered dead if it was not able to move upon being touched with a fine brush.

### 2.4. Toxicities on the Parasitoid T. anastrephae

Adult parasitoid *T. anastrephae*, up to 24 h old, were submitted to an ingestion bioassay for a 24 h period. For this, the insects were deprived of food for 8 h prior to the installation of the bioassays and placed inside plastic cages (100 mL) (10 pairs per cage), as described by Bernardi et al. [30]. The treatments were composed of compound **4** and (*rac*)-8, prepared as described in the “Chemical solutions preparation” section of this article. After 24 h of exposure, the insecticide-contaminated diets were removed, and the insects were provided with pure honey as a food source until the end of the bioassay. Insect mortality was evaluated for up to 120 h following the beginning of exposure, and the data were submitted to a survival analysis on Sigma Plot 12.5 (Systat software Inc., San Jose, CA, USA). The experimental design was completely randomized with seven replicates per treatment, with each replicate being composed of 10 pairs of *T. anastrephae* (n = 140).

To evaluate the sublethal effects of the treatments on the wasps’ functional parasitism abilities, ten *D. suzukii* pupae (24 h old pupae) were offered per day for seven days (beginning at 120 h) to each surviving *T. anastrephae* female from the ingestion bioassay. The pupae were exposed to the wasps on a wet hydrophilic cotton layer on an acrylic petri dish. Daily, the pupae were removed and placed in plastic cups (100 mL) sealed on top with voile until the fly or wasp emerged. During the evaluation period, the wasps were fed with 80% (*w*/*v*) honey/water. The number of parasitoid offspring that emerged was recorded, and the percentage of parasitism was estimated for each treatment during the 7 days of pupae exposure. 

The percentage of parasitism data used for the function of treatment and days of pupae exposure was submitted to a covariance analysis using Proc Mixed in SAS software v 12.0 (SAS Inc. 2013, Cary, NC, USA) with three levels for the first covariable (i.e., control, compound **4** and (*rac*)-8) and seven levels for the second covariable (i.e., first through seventh day). The covariant structure used was compound symmetry based on the smallest AICC (corrected Akaike’s Information Criterion) obtained for this structure among several other covariant structures tested.

### 2.5. In Silico Evaluation of the Potential Target Receptors of Lactone Derivates on D. suzukii and T. anastrephae

#### 2.5.1. Prediction of Putative Targets of Lactone Derivates

The selective lactone molecules in favor of parasitoid insects were drawn using Marvin Sketch 18.12.0 (ChemAxon, Budapest, Hungary) and saved in 3D mol2 format. Target receptor predictions of lactone derivates was carried out with the Similarity Ensemble Approach (SEA) and SwissTargetPrediction databases [31,32]. The genes of the predicted target receptors were downloaded from the NCBI and Uniprot databases and the better interactions against the selective lactone molecules determined from AutoDock Vina software (CCSB, Center for Computational Structural Biology, La Jolla, CA, USA) were used for the further analysis of molecular docking in both spotted wing drosophila and parasitoids.

#### 2.5.2. Data Resources for the Selected Target Receptors of *D. suzukii* and *T. anastrephae*

The amino acid sequences of transient receptor potential (TRP) channels and gamma aminobutyric acid GABA receptors of *D. suzukii* were retrieved from the National Center for Biotechnology Information (NCBI) database. On the other hand, *T. anastrepha* has no sequenced data resource available. Therefore, the proteins of a closely related species, *Trichopria drosophilae*, were selected. The *T. drosophilae* proteins were obtained from the transcriptome data found in the original SRA RNA-seq reads available from the National Center for Biotechnology Information (NCBI). Sequence quality was assessed for each dataset through visualization in FastQC (released 0.11.5). Adapters were removed and low-quality regions were discarded using Trimmomatic (version 0.36). Low-quality readings (mean score of less than 20) and those with less than 50 nucleotides were excluded [33]. After processing the raw readings, we proceeded with their reconstitution through Trinity (version 2.5.1) with the default settings, resulting in contigs of the transcription sequences [34]. Then, we performed the prediction of coding sequences with more than 100 amino acids using TransDecoder [35]. We used Blast2GO to perform a functional annotation with default parameters and an InterProScan analysis of the TransDecoder to predict coding transcripts [36]. After obtaining the GO annotation for every coding transcript, the GABA receptor and TRP channel were identified. Protein domains for both the GABA receptor and TRP channel were identified using HMMER (release 3.0) with the PFAM database.

#### 2.5.3. Generation and Validation of 3D Structures of Target Receptors

Homology modeling was used to construct the 3D structures of both the GABA receptor and TRP channel using The Swiss Model Workspace (https://swissmodel.expasy.org/ accessed on 17 January 2023). The templates were selected using the BLASTp tool, and the crystallographic structures were obtained from the Protein Data Bank (https://www.rcsb.org/ accessed on 17 January 2023). For the choice of the best structures, the experimental method used and the quality parameters (i.e., resolution) considered were the R-value and its complexing with a ligand. Clashes in crystallographic structures and amino acid positioning in the active site were checked using the Swiss model [37]. The validation of the stability of the generated models was performed by analyzing the Ramachandran plot [34,38], in which it was possible to analyze the distribution of the torsion angles of the backbone, Φ and ψ, which are responsible for the stereochemical quality of the protein studies, and the QMEAN factor was also analyzed [39].

#### 2.5.4. Molecular Docking of Lactone Derivates against Target Receptors

Both selective lactone molecules designed by Marvin Sketch 18.12.0 (ChemAxon) and the target receptors modeled were specified to the pdbqt format and were prepared for the molecular docking process using Autodock ps 1.5.7 [40,41]. First, we added hydrogen atoms to the ligands in order to compute the protonation states as well as all possible bond torsions. The coordinates used for docking were generated by positioning the grid box inside the receptor’s active pocket, and the crystallographic structures were used to design the grid boxes. Posteriorly, the docking calculations were performed using AutoDock Vina 4 [42], and nine docking positions for each ligand interacting with all receptors’ active sites were generated. Affinity energies (kcal/Mol) for each interaction were also provided. The results were analyzed using PyMOL 2.0 [43] and Discovery Studio 4.5 [44], and the best interaction positions were selected. The following parameters were used to determine the best positions: ligand interactions with the amino acids from the active site, receptor–ligand affinity energies, the root-mean-square deviation (RMSD) between the initial and subsequent ligand structures and the nature of interactions considering the hydrogen bonds and non-covalent interactions for each complex according to 2D interaction maps.

#### 2.5.5. Phylogenetic Analysis of TRP Channels

The analysis of the evolution of the *D. Suzukii* and *T. anastrephae* TRP channels was conducted using TRP channel genes of seven other species, i.e., *Drosophila melanogaster* (Dm)*, Bombyx mori* (Bm)*, Tribolium castaneum* (Tc)*, Apis mellifera* (Am)*, Nasonia vitripennis* (Nv), and *Pediculus humanus* (Ph) [45]. For this, the sequences were aligned using Muscle software, and the maximum likelihood method was used to calculate the tree based on the WAG amino acid substitution model and with 100 bootstrapped datasets using MEGA6 (Molecular Evolutionary Genetics Analysis) software [46]. The results were visualized and represented using FigTree software (http://tree.bio.ed.ac.uk/software/figtree/ accessed on 17 January 2023). The analysis involved 79 amino acid sequences. The amino acid subfamilies of TRPA (XP_016934147.1–XP_036671523.19) and TRPC (XP_016945947.1–XP_036675292.1) of *Drosophila suzukii* were obtained from the NCBI (National Center and Biotechnological Information). 

## 3. Results

### 3.1. Insecticide Activity of Lactone Derivatives

**Figure 1:**
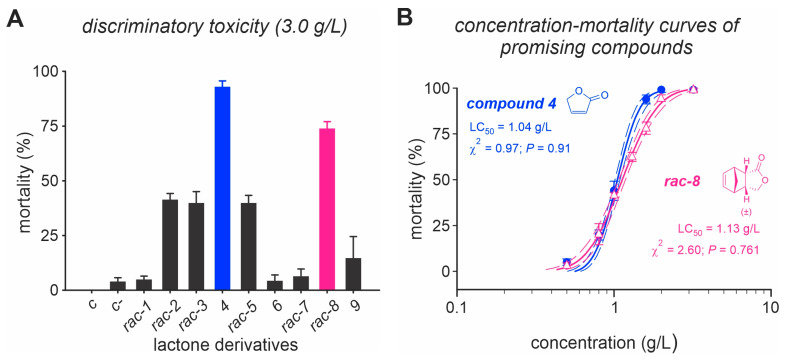
Toxicity screening bioassay of lactone derivatives on *Drosophila suzukii*. (**A**) Mortality of *D. suzukii* adults caused by nine lactone derivatives at a concentration of 3 g/L. (**B**) Concentration–mortality curves for the two most promising molecules (i.e., compound (**4**) and (*rac*)-8). Adult flies were exposed through the ingestion pathway, and the exposure period was 24 h. Control C represents insects treated with sugar solution. Control C represents insects treated with sugar solution containing 2% dimethyl sulfoxide (DMSO).

Lactone derivatives exhibited varying toxicities (F_14,75_ = 48.2, *p* < 0.001) against adult *D. suzukii* (Figure 1A). Among the tested compounds, five molecules, (*rac*)-2, (*rac*)-3, compound **4**, (*rac*)-5 and (*rac*)-8, demonstrated the ability to kill over 40% of *D. suzukii* adults. Compound 4 and (*rac*)-8 displayed the highest potencies with mortality rates exceeding 75% at a concentration of 3 g/L (Figure 1A). However, compound **4** (LC_50_ = 1.04 (1.01–1.08) g/L) and (*rac*)-8 (LC_50_ = 1.13 (1.07–1.18) g/L showed statistically non-significant differences in terms of toxicity (Figure 1B).

### 3.2. Functional Selectivity of Compound 4 and (rac)-8 Lactone Derivates to T. anastrephae Adults

**Figure 2:**
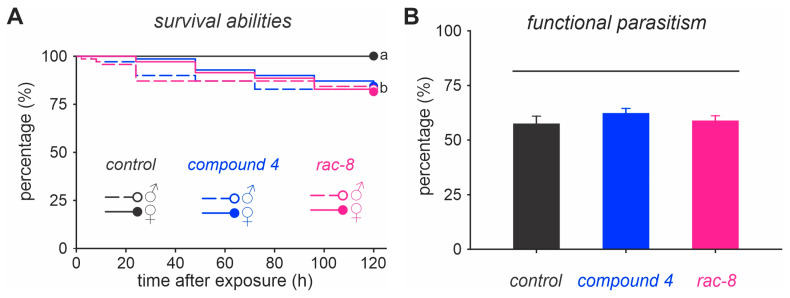
Selectivity of two lactone derivatives (i.e., compound (**4**) and (*rac*)-8) on males and females of the parasitoid *Trichopria anastrephae*. (**A**) Survival of *T. anastrephae* adults exposed to the LC_90_ of compound **4** (1.46 g/L) and (*rac*)-8 (1.91 g/L) estimated for *D. suzukii*. Survival curves followed by the same letter do not differ from each other (log rank test, *p* > 0.05). (**B**) Functional parasitism of *T. anastrephae* females after being exposed to compound **4** (1.46 g/L) and (*rac*)-8 (1.91 g/L). Columns represent the combined daily parasitism rate over a seven-day period after 24 h of exposure to the compounds. Columns under the same horizontal line do not differ from each other (Holm–Sidak test, *p* > 0.05). The control represents insects treated with sugar solution containing 2% dimethyl sulfoxide (DMSO).

The survival analysis of parasitoid males and females indicated that individuals exposed to the estimated LC_90_ for compound **4** (1.46 g/L) and (*rac*)-8 (1.91 g/L) had significantly (*log*-*rank test*, χ^2^ = 27.5, *p* < 0.001) lower survival abilities that those individuals that were not exposed to the lactone derivatives (Figure 2A). However, at the end of the experiment (i.e., 120 h) the survival rate for all exposed insects was greater than 80%. Additionally, exposure to the LC_90_ of lactone derivatives did not affect the ability of *T. anastrephae* to parasitize *D. suzukii* pupae (Figure 2B).

### 3.3. Molecular Docking Analysis of the TRP Channels with Lactone Derivatives

**Figure 3:**
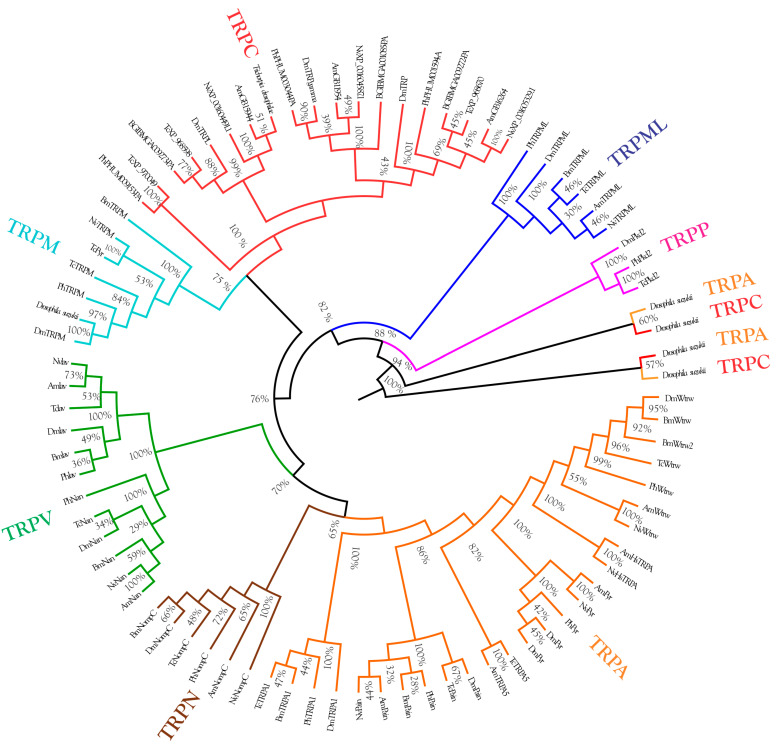
Analysis of the evolution of TRP channels of *Drosophila suzukii* and the *Trichopria drosophilae* species, which is closely related to *Trichopria anastrephae*. The identities of the TRP channels were determined using TRP channel genes of the *Drosophila melanogaster*, *Bombyx mori*, *Tribolium castaneum*, *Apis mellifera*, *Nasonia vitripennis*, and *Pediculus humanus* genomes.

The phylogenetic analysis revealed the evolution of TRP channels of *Drosophila suzukii* and the *Trichopria drosophilae* species, which is closely related to *Trichopria anastrephae* (Figure 3). 

**Figure 4:**
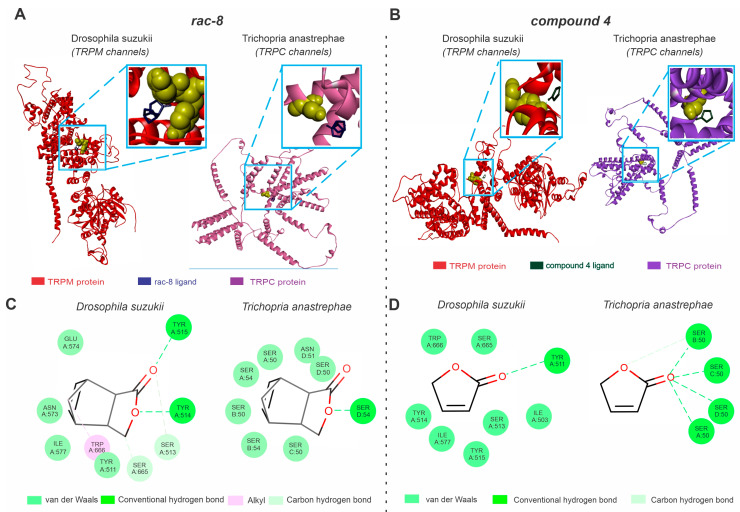
Predictions of (rac-8) and compound **4** lactone derivative binding to TRP channels related to *Drosophila suzukii* and the species closely related to the *Trichopria anastrephae* parasitoid, *Trichopria drosophilae*. (**A**) Structures of active sites of the *Drosophila suzukii* TRP channel (TRPM, left panel) and *Trichopria drosophilae* TRP channels (TRPC, right panel) interacting with (*rac*)-8 (blue). (**B**) Structures of active sites of the *D. suzukii* TRP channel (TRPM, left panel) and *Trichopria drosophilae* TRP channels (TRPC, right panel) interacting with compound **4** (green). (**C**) Two-dimensional interaction map representations of *D. suzukii* and *T. drosophilae* TRP channels with (*rac*)-8. (**D**) Two-dimensional interaction map representations of *D. suzukii* and *T. drosophilae* TRP channels with compound **4**. All detailed amino acids belonging to the lipid environment binding site are also represented. (For the interpretation of the references used to color in this figure legend, the reader is referred to the web version of this article).

Our in silico analysis indicated that the TRPM channels are potential targets for the actions of (*rac*)-8 and compound **4** in *D. suzukii* (Figure 4), but transcriptome analyses did not result in TRPM sequence availability in the parasitoid flies. *T. anastrephae* individuals are equipped with TRPC channels (Figure 4). The TRPM channels exhibited a Ramachandran value of 92.62% and a QMEAN factor of −2.95 (Figure 4A), while the TRPC channels showed similar results for the Ramachandran (91.8%) and QMEAN factor (−4.01) values (Figure 4A).

The complex formed by (*rac*)-8 and the TRPM channels showed hydrogen bond interactions with TYR514 and TYR515, van der Waals interactions with TYR511, ILE577, ASN573 and GLU574, carbon hydrogen bond interactions with SER665 and SER513 and alkyl interactions with TRP666 (Figure 4B). The predicted binding interactions of (rac)-8 with the TRPC revealed higher instability as the predominant forces were van der Waals forces (Figure 4B). The (*rac*)-8 complex with the TRPC channel showed hydrogen bond interactions with SER54 and van der Waals interactions with SER C:50, SER B:54, SER B:50, SER A:54, SER A:50, ASN D:51 and SER D:50 (Figure 4B). Compound 4 exhibited a lower interaction energy (AutoDockVina affinity energy kcal mol^−1^) between the TRPM channel (−3.9) compared to TRPC (−3.2) (Figure 4B). While the SWD-related compound **4** TRPM complex showed hydrogen bond interactions with TYR A: 511 and van de Waals interactions with TRP A: 666, SER A: 665, TYR A: 514, ILE A: 577, TYR A: 515, SER A: 513 and ILE A: 503, the complex formed by compound **4** and TRPC channels showed hydrogen bonds with SER A: 50, SER B: 50, SER C: 50 and SER D: 50 and carbon hydrogen bonds with SER A: 50 (Figure 4B).

### 3.4. Molecular Docking Analysis of the GABA Receptors with Lactone Derivatives

**Figure 5:**
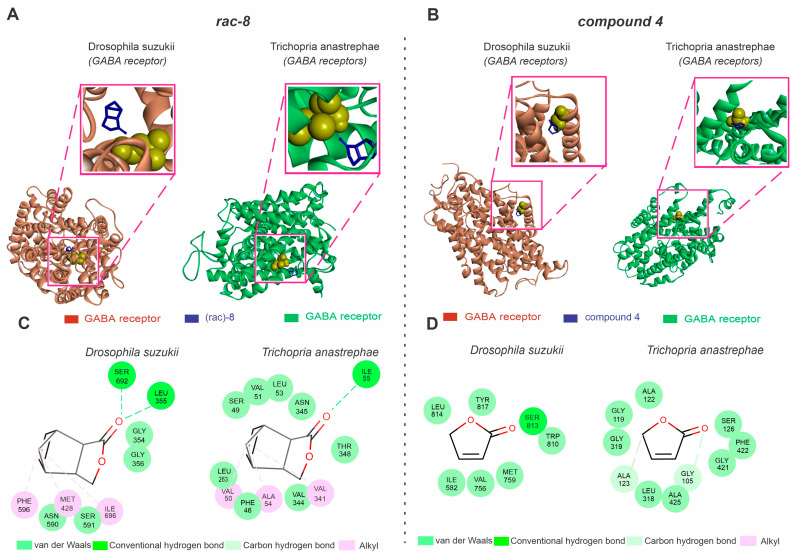
Predictions of (rac-8) and compound **4** lactone derivative binding to the GABA transporter of *Drosophila suzukii* and the species closely related to the *Trichopria anastrephae* parasitoid, *Trichopria drosophilae*. (**A**) Structures of *Drosophila suzukii* (left panel) and *Trichopria drosophilae* (right panel) GABA receptor active sites interacting with (*rac*)-8 (blue). (**B**) Structures of *Drosophila suzukii* (left panel) and *Trichopria drosophilae* (right panel) GABA receptor active sites interacting with compound **4** (blue). (**C**) Two-dimensional interaction map representations of *D. suzukii* and *T. drosophilae* GABA receptors with (*rac*)-8. (**D**) Two-dimensional interaction map representations of *D. suzukii* and *T. drosophilae* GABA receptors with compound **4**. All detailed amino acids belonging to the lipid environment binding site are also represented. (For the interpretation of the references used to color in this figure legend, the reader is referred to the web version of this article).

Our in silico analysis indicated that the GABA receptor is a potential target for the actions of only (*rac*)-8 in *D. suzukii* (Figure 5). The *D. suzukii* GABA receptors exhibited a Ramachandran value of 94.81% and a QMEAN factor of −3.8 (Figure 5A), while GABA receptors of the parasitoids showed similar Ramachandran (92.0%) and QMEAN factor (−3.9) values (Figure 5A). The molecular docking results predicted no significant differences in the interaction energy (AutoDockVina affinity energy kcal mol^−1^) between the GABA receptors of *D. suzukii* (−6.1) and its parasitoid (−5.9) with (*rac*)-8 (Figure 5A). While the (*rac*)-8 complex with the *D. suzukii* GABA receptor showed hydrogen bond interactions with SER692 and LEU355, van de Waals interactions with GLY356, GLY354, SER591 and ASN590 and alkyl interactions with PHE596, MET428 and ILE596, the complex formed by (*rac*)-8 and the parasitoid-related GABA receptors revealed hydrogen bond interactions with ILE55, van der Waals interactions with THR348, VAL344, PHE46, LEU263, SER49, VAL51, LEU53 and ASN345, and alkyl interactions with ALA54. Similarly, the compound **4** complex with *D. suzukii* GABA receptors (−3.9) did not exhibit significant differences in interaction energy (AutoDockVina affinity energy kcal mol^−1^) when compared to the interaction affinity recorded for the GABA receptors of the parasitoids (−4.0). While the compound **4** complex with the *D. suzuki* GABA receptors showed hydrogen bond interactions with SER813, and van der Waals interactions with TRP810, MET759, VAL756, ILE582, LEU814 and TYR817, the compound **4** complex with the parasitoid-related GABA receptors showed carbon hydrogen bond interactions with ALA123 and GLY105 and van der Waals interactions with ALA122, GLY119, GLY319, LEU318, ALA425, GLY421, PHE422 and SER126.

## 4. Discussion

Here, we presented a description of novel lactone-derived molecules that exhibit potential for integration into *D. suzukii* management strategies. We demonstrated that two of these molecules, (*rac*)-8 and compound **4**, possess similar levels of efficacy for the killing of *D. suzukii* adults while leaving the parasitism functionalities of *T. anastrephae* unaffected. Additionally, through molecular docking analysis, we identified the mechanisms by which these molecules interact with the GABA receptors and TRP channels of *D. suzukii* and its parasitoids. This analysis shows that the effectiveness of these molecules against *D. suzukii*, as opposed to *T. anastrephae*, may be attributed to their distinct actions on the TRP channel subtypes present in these insect species.

It is already known that lactone-based compounds have toxic and antifeeding effects on pest insects [47,48,49]. For instance, Szczepanik et al. [48] demonstrated that lactone ring compounds cause feeding inhibition and behavioral deterrence during the larval growth of the lesser mealworm *Alphitobius diaperinus*. These compounds also showed strong antifeedant properties against adult *A. diaperinus*. Similar results have been described for natural lactones against the variegated cutworms *Peridroma saucia* [47] and *Spodoptera frugiperda* [49]. Our efforts reinforce such insecticide activities of lactone derivatives, demonstrating their potential to kill *D. suzukii* adults with lower toxicity and a complete absence of detrimental effects by *T. anastrephae* parasitism to its hosts. It is notable that *T. anastrephae* is one of the most promising biological agents for *D. suzukii* in Neotropical fruit orchards [30].

The potential integration of the (*rac*)-8 and compound **4** lactone derivatives into the management of *D. suzukii* would span a number of effective practices used for controlling *D. suzukii* in the Neotropical region. The reduced number of effective control practices for *D. suzukii* has been a serious problem for cherry and berry production in the Neotropical region [1,2,50,51,52,53]. The reliance on a reduced number of molecules can either be worsened by the fact that some of these molecules can also have detrimental effects on non-target organisms [54,55]. For instance, *T. anastrephae* has been shown to be susceptible to the conventional insecticides used in the management of *D. suzukii* [7,56,57].

Recent investigations that combined in vivo and in silico toxicological approaches have shown that both GABA receptors and TRP channels play relevant roles in the distinct actions of novel insecticides in insect pests and their natural enemies [24,25,27]. Here, our sequence phylogenetic analysis indicated that the *D. suzukii* and *T. anastrephae* might be equipped with different types of TRP channels. While *D. suzukii* expresses the TRPM channel type, which is involved in the removal functions of Mg^2+^ from hemolymph [58], repellent activities [59] and temperature avoidance [60], such channels are not present in *T. anastrephae.* The parasitoid expresses the TRPC channels, which were shown to have less stable molecular interactions in their lipid-binding environment with both (*rac*)-8 and compound **4** in our in silico predictions. This may explain the lower susceptibility of the parasitoid to both lactone derivatives. Interestingly, the stable interactions of both lactone derivatives and GABA receptors of *D. suzukii*, which were also recorded with *T. anastrephae* GABA receptors, did not allow the identification of the GABA receptors as a potential reason for the selectivity of (*rac*)-8 and compound **4** towards the parasitoid. 

## 5. Conclusions

Despite further investigation aiming to evaluate further steps necessary to develop a pesticide product (e.g., formulation type, application method and evaluations of efficacy at field conditions), our findings represent a relevant and promising step that could lead to the development of novel tools for controlling *D. suzukii*. Our investigations demonstrate that lactone-derived molecules, (*rac*)-8 and compound **4** can effectively kill *D. suzukii* by targeting TRP channels and GABA receptors. Notably, these lactone derivatives exhibit reduced toxicity towards *T. anastrephae* with no adverse effects on functional parasitism. This selective efficacy against *D. suzukii* can be attributed to the expression of a specific TRP channel type (TRPM) in the fly, which facilitates more stable molecular interactions compared to the TRP channels expressed in the parasitoid (TRPC). Furthermore, the lactone derivatives’ actions on GABA receptors were comparable in both insect species and thus do not contribute to the explanation of the lactone derivative’s selectivity. Our findings demonstrate that both (*rac*)-8 and compound **4** exhibit the potential to be integrated into *D. suzukii* management. 

## Data Availability

The data presented in this study are available on request from the corresponding authors.

## Supplementary Materials

The following supporting information can be downloaded at: https://www.mdpi.com/article/10.3390/insects14080697/s1, Table S1: Lactone derivative compounds.
