# Supplementary material for: Novel Lactone-Based Insecticides and Drosophila suzukii Management: Synthesis, Potential Action Mechanisms and Selectivity for Non-Target Parasitoids
Source: Insects. 2023 Aug 9;14(8):697. doi: 10.3390/insects14080697 (PMC10455539; doi:10.3390/insects14080697)
Supplement: Supplementary file 1 [file insects-14-00697-s001.zip › insects-2479006-supplementary.pdf]

Supplementary Table S1. Lactone derivative compounds.

Molecule Identification[1]	Molecular Structure	Molecule Name
(rac)-1	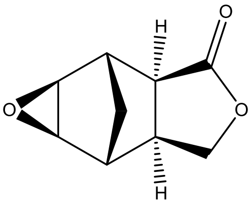	(3aS,4R,5R,6S,7S,7aS)- and (3aR,4S,5S,6R,7R,7aR)-5,6-epoxyhexahydro-4,7-methanoisobenzofuran-1(3H)-one
(rac)-2	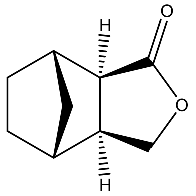	(3aS,4R,7S,7aR)- and (3aR,4S,7R,7aS)-hexahydro-4,7-methanoisobenzofuran-1(3H)-one
(rac)-3	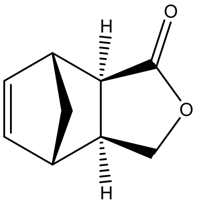	(3aS,4S,7R,7aR)- and (3aR,4R,7S,7aS)-3a,4,7,7a-tetrahydro-4,7-methanoisobenzofuran-1(3H)-one
4	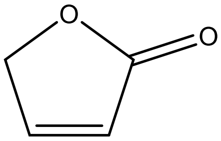	furan-2(5H)-one
(rac)-5	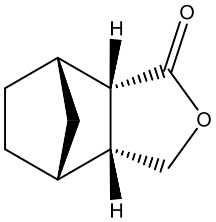	(3aR,4R,7S,7aS)- and (3aS,4S,7R,7aR)-hexahydro-4,7-methanoisobenzofuran-1(3H)-one
6	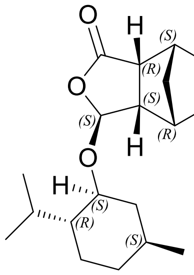	(3S,3aS,4R,7S,7aR)-3-(((1S,2R,5S)-2-isopropyl-5-methylcyclohexyl)oxy)-3a,4,7,7a-tetrahydro-4,7-methanoisobenzofuran-1(3H)-one
(rac)-7	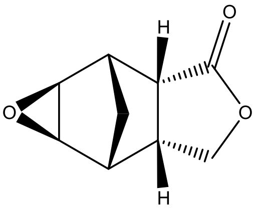	(3aR,4R,5R,6S,7S,7aR)- and (3aS,4S,5S,6R,7R,7aS)-5,6-epoxyhexahydro-4,7-methanoisobenzofuran-1(3H)-one

(rac)-8	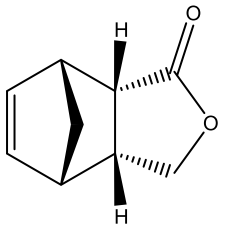	(3aR,4S,7R,7aS)- and (3aS,4R,7S,7aR)-3a,4,7,7a-tetrahydro-4,7 methanoisobenzofuran-1(3H)-one
9	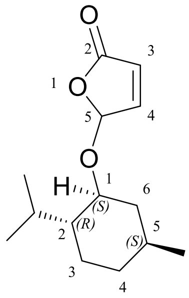	5-(((1S,2R,5S)-2-isopropyl-5-methylcyclohexyl)oxy)furan-2(5H)-one

[1] All compounds named (rac) are racemic mixtures.
